# A *Drosophila melanogaster* model shows that fast growing *Metarhizium* species are the deadliest despite eliciting a strong immune response

**DOI:** 10.1080/21505594.2023.2275493

**Published:** 2023-11-08

**Authors:** Jonathan B. Wang, Hsiao-Ling Lu, Huiyu Sheng, Raymond J St. Leger

**Affiliations:** Department of Entomology, University of Maryland, College Park, MD, USA

**Keywords:** *Drosophila*, resistance and tolerance; *metarhizium*, immune activation; immunity, gender differences; Bomanins, inhibit *metarhizium*; Persephone, fungal loads

## Abstract

We used *Drosophila melanogaster* to investigate how differences between *Metarhizium* species in growth rate and mechanisms of pathogenesis influence the outcome of infection. We found that the most rapid germinators and growers *in vitro* and on fly cuticle were the fastest killers, suggesting that pre-penetration competence is key to *Metarhizium* success. Virulent strains also induced the largest immune response, which did not depend on profuse growth within hosts as virulent toxin-producing strains only proliferated post-mortem while slow-killing strains that were specialized to other insects grew profusely pre-mortem. *Metarhizium* strains have apparently evolved resistance to widely distributed defenses such as the defensin Toll product drosomycin, but they were inhibited by Bomanins only found in *Drosophila* spp. Disrupting a gene (*Dif*), that mediates Toll immunity has little impact on the lethality of most *Metarhizium* strains (an exception being the early diverged *M. frigidum* and another insect pathogen *Beauveria bassiana*). However, disrupting the sensor of fungal proteases (*Persephone*) allowed rapid proliferation of strains within hosts (with the exception of *M. album*), and flies succumbed rapidly. Persephone also mediates gender differences in immune responses that determine whether male or female flies die sooner. We conclude that some strain differences in growth within hosts depend on immune-mediated interactions but intrinsic differences in pathogenic mechanisms are more important. Thus, *Drosophila* varies greatly in tolerance to different *Metarhizium* strains, in part because some of them produce toxins. Our results further develop *D. melanogaster* as a tractable model system for understanding insect-*Metarhizium* interactions.

## Introduction

Most models of interactions between hosts and pathogens are based on the concept of tightly coupled, co-evolved interactions between species pairs [1]. This is despite the fact that most pathogens of plants and animals are generalists that infect multiple-host species and evidence that many emerging diseases are caused by generalists, of which fungal diseases make up the majority [2]. The outcomes of pathogen infection vary widely because hosts differ in their resistance and tolerance to infection, while pathogens vary in their ability to grow on or within hosts [3]. This variation determines the burden of disease and represents the raw material from which populations can evolve resistance [4]. Insects are continually exposed to a vast number of potential pathogens, and they have evolved a series of intricate mechanisms to resist pathogen attacks [5]. These pathogens are dynamic agents of host selection as reflected in the genetic variation in resistance in wild insect populations [6–8].

Fungi cause a large proportion of insect diseases [9] and include the ascomycete genus *Metarhizium*, which is a radiating lineage of insect pathogens [10]. In addition to their crucial role in natural ecosystems, *Metarhizium* spp. are frequently used as biological insecticides [11,12] and for genomic studies on the nature of adaptive differences by which novel pathogens emerge and form new species [13]. While many *Metarhizium* strains in nature seem to be limited to a narrow range of hosts, others attack a broad range of species. Thus, *Metarhizium album, Metarhizium acridum* and *Metarhizium majus* specialize in hemipterans, orthopterans, and coleopteran insects, respectively [14,15]. *Metarhizium frigidum* has evolved independently as a generalist, whereas *Metarhizium pingshaense, Metarhizium anisopliae*, *Metarhizium robertsii,* and *Metarhizium brunneum* (the PARB clade) have more recently evolved and parasitize many insect orders, including dipterans [16,17]. *Metarhizium* spp. thus provide a model for studying the basis of generalism and specificity and the potential of pathogens to cross the species barrier and infect new hosts.

An infection by *Metarhizium* typically starts with conidial adhesion to the insect integument, followed by germination, which is triggered by topographical and chemical signals from insect cuticles and environmental cues, such as relative humidity [18,19]. Germlings produce adhesive infection structures (appressoria), and hyphal penetration through the host cuticle occurs through a combination of mechanical pressure and cuticle-degrading enzymes including many proteases [20,21]. Penetrating multicellular hyphae respond to factors present in the host hemolymph by switching to growth as single-celled blastospores that facilitate dissemination and have mechanisms to evade the insect immune system [19,22]. Once the host is dead, the fungus breaches the cuticle from the inside outwards, allowing the formation of conidia that, upon dispersal, start new infections [21]. Thus, the onward transmission of *Metarhizium* requires the death of the host, that is, it is an obligate killer.

Some *Metarhizium* strains are capable of infecting *Drosophila melanogaster* providing a genetically tractable system for studying host–pathogen interactions. Those strains that are not adapted to *Drosophila* are presumably unable to infect, grow within the fly, or transmit to new hosts as well-adapted pathogens. Relating host specificity and infection, *Metarhizium* species differ in the host-related factors required to induce appressoria [23], and in their infection strategies. For example, both *M. anisopliae* ARSEF strain 549 (Ma549) and *M. robertsii* ARSEF strain 2575 (Mr2575) have broad host ranges, but Ma549 is biotrophic (grows through the living host) and produces little destruxins (toxins) whereas Mr2575 kills with toxins and is subsequently necrotrophic [24]. Like Ma549, the broad host range *M. frigidum* also lacks destruxins, although in general nontoxigenic *Metarhizium*, spp. such as *M. album, M. acridum,* and *M. majus* have narrow host ranges [15].

Fungal infection processes encounter a dedicated immune response that includes melanization and antimicrobial peptides (AMPs) [5], generated by the highly conserved Toll pathway, the chief *D. melanogaster* antifungal pathway described in the literature [25,26]. In this species, the circulating protease Persephone (*Psh*), an immune sensor of pathogen proteases, and GNBP3, which detects fungal wall components, act exclusively to detect infection. They link their activation to proteolytic serine protease cascades, which induce Toll-mediated AMP transcription through nuclear translocation of *Drosophila* Dif, an NF-κB homolog [27,28]. Insects disrupted in *Dif* or *Psh* succumb quickly to the entomopathogenic fungus *Beauveria bassiana* [29]. We previously found that despite fungal recognition and Toll immune elicitation by flies, infection with Ma549 could not be successfully eliminated [30]. A genome-wide association study (GWAS) deploying the *Drosophila* Genetic Reference Panel (DGRP) found considerable genetic variation in the susceptibility of *D*. *melanogaster* to Ma549, but how long DGRP lines took to succumb to Ma549 was not associated with differences in genes implicated in canonical immune processes [7]. These studies have revealed a complex genetic architecture for disease resistance to Ma549, with large numbers of pleiotropic genes and alleles with sex-, environment-, and genetic background-specific effects. The extent to which the resistance architecture of *Drosophila* differs against different *Metarhizium* strains is unclear.

**Figure 1.**
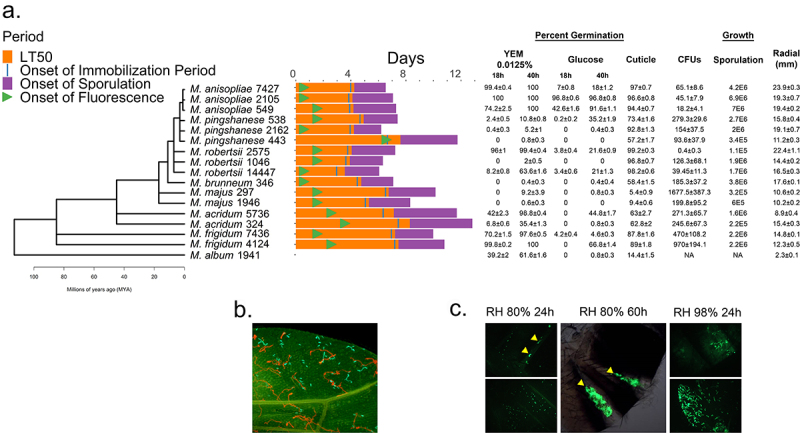
Phylogeny of *Metarhizium* spp. (a) a phylogenomic tree for the *Metarhizium* species used in this study. Right of tree, the bars indicate times for the onset of a fly’s immune response (detected by Drs-GFP fluorescence), immobilization and death. The % germination provided are after 18 or 40 hrs in yeast extract medium (YEM) or in glucose medium, and after 16 hrs on insect cuticle (fly wings). Fungal growth in the hemolymph (colony forming units (CFU’s) at the commencement of the immobilization period), sporulation (spore counts per cadaver) and radial growth on PDA are provided as measures of growth. Further details are provided in Supplemental tables S1 and S2. (b) rapid germination and growth of *M. robertsii* 2575 conidia (expressing cherry) compared to *M. majus* 1946 (expressing GFP) on fly wings (the ungerminated conidia of *M. majus* are approximately 10 µm long) (c) GFP-expressing *M. robertsii* 2105 photographed on the abdomen and wings of a fly 24- and 60-hours post-infection. Flies or their wings were visualized with epifluorescence, with filters set to detect GFP or cherry fluorescence.

In this study, we used a strain of *M. robertsii* isolated from *Drosophila suzukii*, a generalist strain of *Metarhizium* spp. with diverse pathogenic strategies, and specialists not adapted to *Drosophila*, in comparative analyses to examine the relationship between virulence, growth, nutritional plasticity, transmission, and mechanisms to evade host immunity. We tested whether virulence was positively correlated with pathogen growth rate and transmission, as commonly assumed by virulence theory [31], and examined whether virulence was correlated with life-history variables that influence the progression of pathogenesis (Figure 1, Supplementary Table S1). These variables included: (i) the spore dose required to establish infection; (ii) the impact of humidity on infection as humidity is particularly critical for fungal sporulation, germination, and invasion of insect hosts [32]; (iii) the time of onset, magnitude, and efficacy of the Toll immune response; (iv) the period of time preceding death when the host is immobilized (immobilization time); (v) the interval between death and the onset of sporulation; and (vi) sporulation capacity per cadaver. We found that the most virulent strains elicited the strongest immune responses. Therefore, their success as pathogens may be due to their ability to adapt to and resist the insect innate immune response. To investigate this possibility, we surveyed *Metarhizium* strains against *Drosophila* mutants with defects in the immune system. Overall, our analysis indicated that the outcome of an infection depends on factors specific to each pathogen interacting with diverse aspects of host immunity and that there are no simple extrapolations between the magnitude of an immune response and duration of survival, even between related pathogens and a single host species.

## Results

### Infection protocols

Figure 1a shows the phylogeny of the *Metarhizium* strains deployed in this study and the distribution of experimentally determined phenotypes. Further details, including their USDA ARSEF collection accession numbers, original hosts, and infection parameters, including differences in LT_50_, are shown in Supplementary Table S1. Infection involved immersing flies in a spore suspension, typically 2.5 × 10^4^ per ml for studies with mutant flies, which results in ~200 spores/fly [7]. At a high spore concentration (1 × 10^6^ per ml), LT_50_’s ranged from 2.92 (*M. robertsii* 2575) to 5.72 (*M. acridum* 5736). Infection with *M. album* 1941 did not reduce the fly lifespan.

Laboratory infection of *Drosophila* by the “natural route” through the cuticle rather than injection typically involves rolling flies on a plate of sporulating fungi, so the insects are covered in a layer of spores. Taylor and Kimbrell [33] reported that after infection with *B. bassiana* spores in this manner, all parts of the body are groomed and cleaned as much as possible, leaving only the areas that are hard to reach, mainly the back of the thorax, with any visible fungal spores. Our alternative procedure of immersing flies in a spore suspension did not produce a visible layer of spores on the insect. However, using GFP-expressing spores of various *Metarhizium* strains, we found that grooming removed most, but not all, spores from the sclerites (smooth and hard portions of the fly’s body); although, irrespective of the strain, spores were frequently trapped in loose aggregations in the intersegmental regions of the abdomen (Figure 1c). Therefore, grooming is unlikely to contribute to the differential virulence of *Metarhizium* strains.

### How does environmental humidity affect the lethality of *Metarhizium* strains with different virulence?

**Figure 2.**
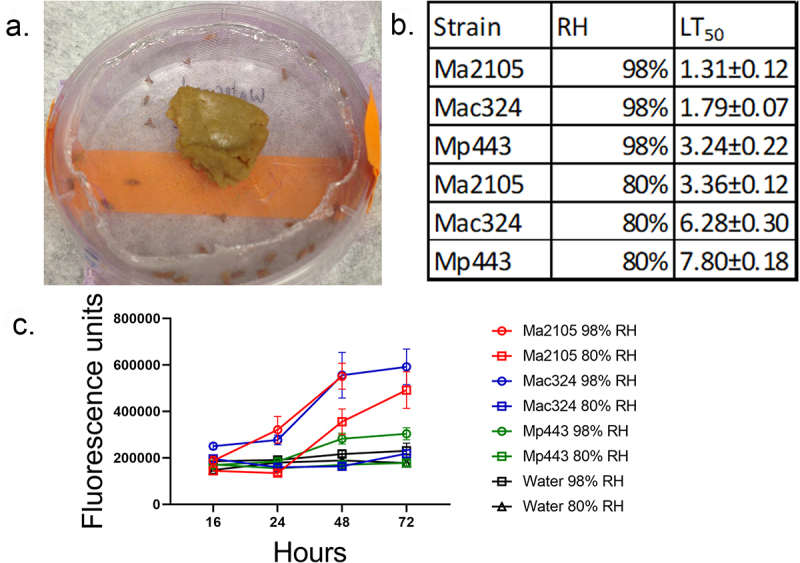
The effect of humidity on *Metarhizium* spp. Fly (Drs-GFP) immune system responses to infection with 5×10^6^ spores of *M. anisopliae* 2015, *M. acridum* 324 and *M. pingshaense* 443 at different relative humidity’s (98%, and 80%) was studied by measuring drosomycin expression and calculating LT_50_ values. a) control and infected flies were maintained in petri dishes covered in nylon mesh (pink) and with access to food placed on the mesh. The petri dishes were placed on water saturated tissues (98% RH) or over a saturated solution of NaCl (80% RH). b) faster kills at higher RH elicits earlier and higher Drs-GFP immunofluorescence in infected insects. Fluorescence data was collected 16, 24, 48, and 72 h post-infection. Points represent the means of 10 individual flies±SE. Control flies were treated with water instead of spore suspensions and incubated at different RH in parallel with infected flies.

We investigated whether favorable humidity for spore germination and pathogenicity was involved in the differential virulence of the strains. We used Toll activity readout (Drs-GFP) flies [containing a reporter construct that expressed GFP (green fluorescent protein) under control of the drosomycin reporter] to check if there was a time difference for immune-response fluorescence at 98% and 80% relative humidity (RH) when infected with representative virulent *M. anisopliae* (Ma2105), intermediate virulence *M. acridum* (Mac324), and low-virulence *M. pingshaense* (Mp443) strains. All three fungal strains killed significantly faster at 98% RH than at 80% RH (*p* < 0.05) (Figure 2). At each RH, Ma2105 was significantly more virulent than Ma324 or Mp443 (At 98% RH, Ma2105 vs. Ma324, *t* = 3.54, *p* = 0.0403; Ma2105 vs Mp443, *t* = 7.61 *p* = 0.0046. At 80% RH, Ma2105 vs. Ma324, *t* = 9.02, *p* = 0.005; Ma2105 vs Mp443, *t* = 36.46 *p* = 0.0004). Increased spore germination at high RH produced an earlier immune response (Figure 2), consistent with more rapid penetration into the insect as Drs-GFP immunofluorescence was negatively correlated with speed of kill (*r* = −0.782, *p* = 0.0128 at 72 h post-infection). Although 98% humidity dramatically increased both mortality and Drs-GFP immunofluorescence for the three strains, it did not make Mp443 or Mac324 as virulent to flies as Ma2105.

Using a high (1 × 10^6^ per ml) spore concentration to provide a sufficient number of spores to count on cuticle surfaces, we also monitored the germination of GFP-fluorescent Mp443 or Ma2105 on different parts of *Drosophila* bodies at different RH levels (Table 1). At 80% RH, germination occurred almost exclusively on intersegmental abdominal membranes, whereas after 16 h at 98% humidity, more than 50% of spores had germinated on all parts of the fly’s body. We conclude that *Drosophila* intersegmental membranes possess an adequate microclimate to promote germination at low ambient RH, as has been reported for larger insects [34].

**Table 1.** The germination rates of two *Metarhizium* strains on *Drosophila melanogaster* at different relative humidity.

		98% RH	80% RH
Hours post-infection	Location	Germination rates (%) (mean±SEM)	Germination rates (%) (mean±SEM)
**Strain Mp443**			
16 hr	Ventral abdomen	59.9±8.06	0
	Dorsal abdomen intersegmental	75.1±4.35	19.6±7.12
	Dorsal abdomen segments	66.8±3.56	0
24 hr	Ventral abdomen	85.2±5.93	0
	Dorsal abdomen intersegmental	88.1±2.83	17.4±1.81
	Dorsal abdomen segments	68.6±5.12	0
48 hr	Ventral abdomen	62.1±0.30	0
	Dorsal abdomen intersegmental	overgrown	81.1±3.85
	Dorsal abdomen segments	50.8±10.77	0
**Strain Mr2105**			
16 hr	Ventral abdomen	65.3±7.88	0
	Dorsal abdomen intersegmental	90.9±1.17	29.2±4.19
	Dorsal abdomen segments	62.4±3.61	0
24 hr	Ventral abdomen	81.3±5.20	0
	Dorsal abdomen intersegmental	91.7±2.91	35.7±8.63
	Dorsal abdomen segments	66.9±12.05	0
48 hr	Ventral abdomen	overgrown	2.5±2.50
	Dorsal abdomen intersegmental	overgrown	overgrown
	Dorsal abdomen segments	overgrown	0

### How do pathogen genotypes with different infection strategies interact with *Drosophila melanogaster*?

Comparing all *Metarhizium* strains, there were strong correlations (r > 0.75, p > 0.0008) between lethal spore doses needed to kill 50% of the flies (LC_50_), and the median lethal time to kill 50% (LT_50_) at three spore doses (1×10^6^, 1 × 10^5^ and 1 × 10^4^ spores per ml), confirming that the fastest killers are also those effective at the lowest spore doses. Spore production on cadavers is a measure of pathogen transmission potential, and therefore, pathogen fitness [30]. The pathogen genotype affected the timing of onset of sporulation on cadavers, which also correlated with immobilization time (a period of pre-mortem low mobility) (r = 0.74, p = 0.001), and values for LC_50_ (r = 0.77, p = 0.0005) and LT_50_ (1 × 10^6^, r = 0.69, p = 0.0031). Although spore production per cadaver was negatively associated with the onset of sporulation (*r* =- 0.37), that is, early sporulators were heavy sporulators, this was not statistically significant (p = 0.1644) (Supplementary Table S1).

Virulence was linked to the host range when we grouped strains into either *M. frigidum* and PARB clade (minus Mp443) generalists or presumed specialists (species associated with a narrow range of hosts in nature). There was a significant difference in LT_50_ values (Welch’s t-test, *t* = 4.39, *p* = 0.0007) between generalist (µ = 3.81 ± 0.257 days) and specialist (µ = 5.25 ± 0.202 days) strains. The hemipteran specialist *M. album* 1941 (Mal1941) did not immobilize or kill the flies. Flies infected with *M. majus* (297 and 1946), *M. acridum* (Mac324 and 5736), and *M. pingshaense* (Mp443) had an extended immobilization time (µ = 15.6 ± 3.70 h.) compared to generalists (µ = 8.18 ± 0.71 h.). However, the difference was not significant (*t* = 1.97, *p* = 0.1152). The 7.4-h average difference in immobilization time accounted for ~6.2% of the longer LT_50_ values delivered by *M. pingshaense* 443 and *M. majus* 297. The hyphae of generalists take significantly less time to emerge from *Drosophila* cadavers (emergent period) than specialists (generalists µ = 36.21 ± 3.32 h; specialists µ = 59.23 ± 8.21 h) (*t* = 2.6, *p* = 0.045). Generalists sporulated faster (µ = 63.99 ± 3.12 h) than specialists (µ = 94.42 ± 7.58 h, *t* = 3.71, *p* = 0.012) and produce more spores (µ = 3.16 × 10^6^ ± 6.46 × 10^5^ spores) than specialists (µ = 6.21 × 10^5^ ± 2.60 × 10^5^ spores) (*t* = 3.65, *p* = 0.0031).

The saprophytic growth of each *Metarhizium* line was estimated from the radial growth of colonies on potato dextrose agar (PDA). Fast growing strains on PDA also germinated faster on *Drosophila* wings (e.g. host cuticle, Figure 1a) (*r* = 0.67, *p* = 0.0046) and killed faster, as shown by the negative correlation with LT_50_ values (spore dose 1 × 10^6^ spores/ml, *r* = −0.75, *p* = 0.0009). Fast growers on PDA sporulated faster on cadavers (*r* = −0.54, *p* = 0.0313), but the association with higher sporulation capacity fell short of significance (*r* = 0.49, *p* = 0.0531) (Supplementary Table S2). We previously characterized variations in metabolic flexibility between different *Metarhizium* strains based on their ability to germinate in yeast extract media (YEM) or on glucose and sodium nitrate as sole carbon and nitrogen sources and to produce appressoria *in vitro* against a hard hydrophobic surface [23]. We repeated these experiments with the strains used in this study. As shown in Figure 1 and Supplementary Table S1, five of the strains were metabolically restricted, showing little or no growth in glucose or a low concentration (0.0125%) of YEM. However, of the five metabolically restricted strains, *M. robertsii* 1046 and *M. pingshaense* 2162 killed flies rapidly, whereas *M. pingshaense* 443 and two *M. majus* were weakly virulent (S Table 1, Figure 1). Two of the *Metarhizium* lines tested, *M. anisopliae* 2105 and *M. robertsii* 14447, were collected from close *D. melanogaster* relations, *Hydrelli* spp., and *D. suzukii*, respectively. Unlike Mr14447, glucose promoted the germination of Ma2105 conidia and even allowed the formation of appressoria. Mr14447 produced appressoria only in 0.01% YEM. Similar to many other lines isolated from scarabaeid coleopterans [23], *M. robertsii* 1046 did not germinate readily on YEM or glucose but killed *D. melanogaster* at a rate similar to that of Mr14447 and the metabolically flexible *M. robertsii* 2575 (Table S1, Figure 1a). *M. robertsii* 2575 (Mr2575) also grew approximately 30% faster than Mr1046 on PDA, but both strains exhibited more than 90% germination on fly wings. Thus, virulence does not depend on whether a strain has broad or narrow nutrient requirements for germination and growth *in vitro*. While fast radial growth on PDA is a reliable predictor of rapid germination on the cuticle and virulence, slower growers on PDA may also be fast killers if they can germinate rapidly on fly wings (Figure 1a). All rapid (>90% in 14 h) germinators on fly wings killed quickly, suggesting that pre-penetration competence is key to *Metarhizium* success.

We previously reported that lines of the *Drosophila melanogaster* Genetic Reference Panel (DGRP) show significant genetic variation in their ability to tolerate Ma549 colonizing their hemolymphs [7]. It is also conceivable that *Drosophila* lines are better able to tolerate some *Metarhizium* strains colonizing the hemolymph than others. We looked for colony forming units (CFUs) in the hemolymph of flies infected with each *Metarhizium* strain at the start of the immobilization period (which varied between strains) (Figure 1a, Supplementary Table S1). Negative correlations between CFU counts and growth on PDA (*r* = −0.5, *p* = 0.0489) or germination on the host cuticle (*r* = −0.54, *p* = 0.03) (Supplementary Table S2) indicated that slow-growing strains on PDA and cuticle tended to proliferate abundantly in the hemolymph. Thus, most flies infected with the virulent Mr2575 did not show CFUs until post-mortem, whereas the slow-killing *M. majus* Mm297 produced 1677.5 ± 387.3 CFUs per fly at immobilization time.

Generalist strains are often toxigenic, producing destruxins (dtxs), whereas nontoxigenic *Metarhizium* spp. (e.g. *M. acridum, M. majus, M. album*) usually have narrow host ranges and kill by growing within the host [15]. Destruxins suppress immune responses to facilitate fungal colonization in insects [15,35]. Mr2575 produces high levels of dtxs, even when colonizing plants [36], suggesting that their production is constitutive. Mr2575 grew well on PDA and fly cuticle, suggesting that toxin production does not necessarily compete with metabolic processes associated with growth. We used an Mr2575 null mutant of dtxs [37] to test whether dtxs contribute to the short immobilization period. A Kruskal–Wallis test showed no significant differences (χ2 = 0.19, *p* = 0.91) in the immobilization period of Drs-GFP flies infected with 2575ΔDtx and Mr2575, pointing to the complexity of virulence determination beyond individual toxic metabolites.

### *Drosophila* immune response to different *Metarhizium* strains.

**Figure 3.**
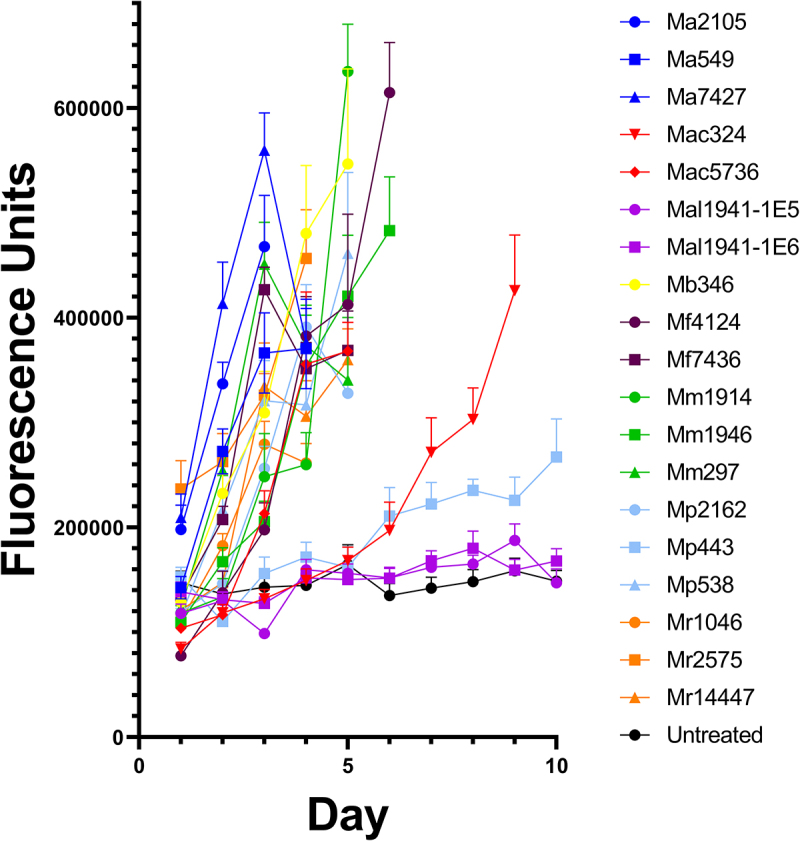
Drs-GFP immunofluorescence response of *D. melanogaster* to infection with different *Metarhizium* strains of high, medium and low virulence to *Drosophila.*

We next hypothesized that immune activity might play a role in variation in pathogen success. Drs-GFP flies were used to measure immune activation. There was an association between a strain’s virulence and the induction of drosomycin, as illustrated by the time course of GFP immunofluorescence in Drs-GFP flies (Figure 3). Low-virulence strains that germinated poorly on the cuticle-induced fluorescence later than fast killers (Figure 3), suggesting delayed penetration and activation of the immune system, as indicated by the positive relationship between LT_50_ and the onset of immunofluorescence (*r* = 0.71, *p* = 0.002). The intensity of the immune response was not significantly correlated with LT_50_s (*r* = 0.32, *p* = 0.22) and the propensity for generalist strains, which usually kill faster, to produce greater maximum immunofluorescence (µ = 4.64 × 10^5^ ± 3.12 × 10^4^) compared to specialists (µ = 3.63 × 10^5^ ± 4.69 × 10^4^) also falls short of significance (*t* = 1.78, *p* = .011). However, the very slow killing *M. pingshaense* 443 produced late and weak immunofluorescence; thus, by day 4, immunofluorescence was more than two standard deviations below flies infected with other fungi (Figure 3).

**Figure 4.**
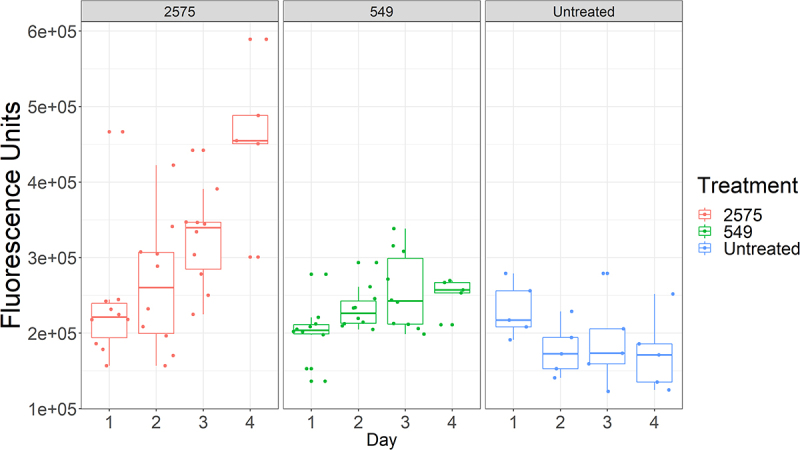
Boxplots showing the variability in immunofluorescence between individual Drs-GFP flies infected with either Ma549 and 2575.

Although virulent strains trigger early detection by the host immune system, there is substantial variation between individual flies in their drosomycin expression for each pathogen. Thus, on day 2, the rapid killers Ma549 and Mr2575 produced approximately 1.4- and 2.7-fold differences, respectively, in extremes of low to high fluorescence (Figure 4). In non-destructive experiments using a fluorescence microscope, we found that ~80% of Drs-GFP flies infected with Ma549 fluoresced by day 2, and the remaining flies began fluorescing after 2.5 days. However, we found no significant differences in longevity between early- and late-fluorescing flies.

Collectively, our results show a link between proliferation of CFUs in the hemolymph and slow speed of kill, and a strong positive association between late drosomycin expression and late mortality, that is, slow killing strains proliferate in the hemocoel and yet induce a late immune response. Neither immune response nor virulence is dependent on extensive fungal proliferation in the hemocoel which suggests that penetration through the cuticle induces the immune response. Thus, Mr2575 and the metabolically restricted Mr1046 were similar in both virulence and timing of drosomycin induction, although Mr2575 produced very few CFU (<1 per insect) before host death, and Mr1046 infected flies had CFU counts >100 (Figure 1). Our results are consistent with virulent strains inducing an earlier immune response of greater magnitude than less virulent strains, showing that virulence does not depend on suppressing a strong immune response.

### Psh-dependent processes and Bomanin peptides confer resistance to *Metarhizium* strains post cuticle penetration

**Figure 5.**
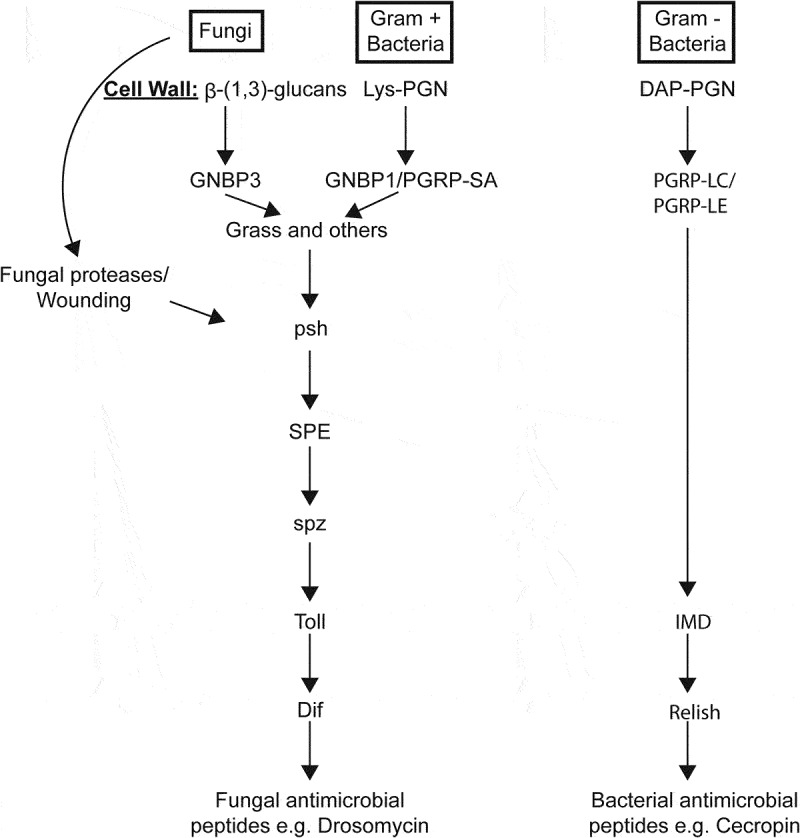
A simplified model of the immune (Toll and Imd) pathways, principally based on Dudzic et al. [38].

To obtain a broader understanding of how successful pathogens adapt to and resist the insect innate immune response, we surveyed *Metarhizium* strains against *Drosophila* mutants with defects in the immune system. A model showing the potential interconnections of the components of Toll and Imd responses represented by the mutants is shown in Figure 5. Flies, either wild type or immune deficiency mutants, were also challenged with *B. bassiana* 80.2, a strain that has been used in several previous studies to screen defective *Drosophila* mutants, thus facilitating direct comparisons between these earlier studies and *Metarhizium* spp. Flies lacking Persephone (*Psh^−^*), a serine protease implicated in the recognition of pathogen proteases [29], succumbed to most *Metarhizium* strains in approximately half the time as their isogenic background. The acridid specialist strain Mac324 killed *Psh^−^* flies ~6 days earlier than the WT, indicating that even some specialists can overcome a non-natural host insect with a key defect in the immune system. However, the low virulence of Mp443 was not improved by the loss of Persephone, and both the WT (w[1118]^6326^) and *Psh^−^* were still alive 10 days post-infection with Mal1941.

**Figure 6.**
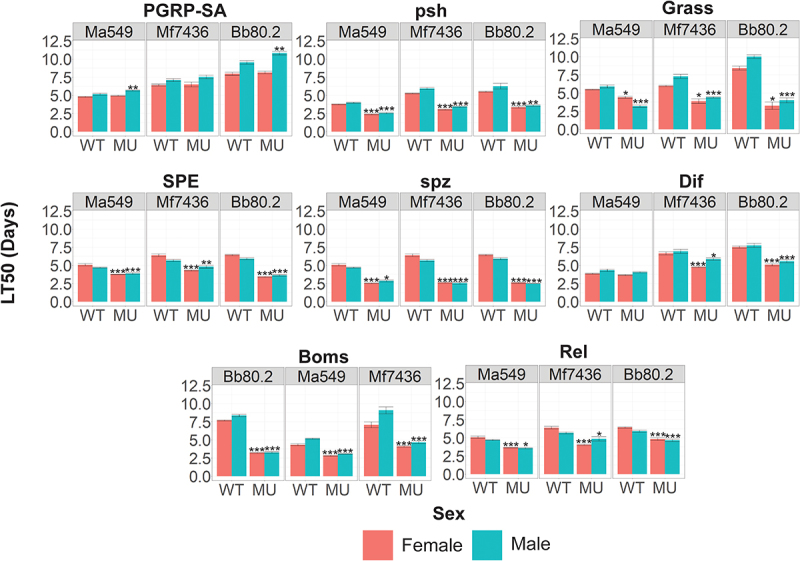
Quantifying the role that individual components of the insect immune system play in resisting infection. survival (measured as LT_50_’s) of fly lines disrupted in known immune genes and their isogenic WT backgrounds against *M. anisopliae* (Ma549), *M. frigidum* (Mf7436) or *B. bassiana* (Bb80.2). Shown is the combination of three independent experiments for each pathogen with ~ 20 flies per genotype per experiment. Significance was evaluated using t-tests and is shown relative to the WT (***p < 0.001; **p < 0.01; *p < 0.05).

Persephone activates a serine protease cascade that induces Toll-mediated AMP transcription through nuclear translocation of *Drosophila* DIF, an NF-κB homolog [27]. However, flies lacking Dif (*Dif* ^−^) succumbed to most *Metarhizium* strains at approximately the same time as the background controls (Figure 6). An exception was *M. frigidum* 7436, in which the survival time of male (female) *Dif* ^−^ flies was 85% (72%) of that of WT flies (Figure 6), *t* = 3.36, *p* = 0.014 (*t* = 7.89, *p* = 0.0007). Similar results were obtained with *B. bassiana* Bb 80.2: the survival time of male (female) *Dif* ^−^flies was 72% (68%) of that of WT flies, *t* = 6.33, *p* = 0.0003 (*t* = 11.28, *p* = 3.43e-6) (Figure 6). These results are in close agreement with earlier studies on Bb 80.2, but according to Le Bourg [39], the appearance of high mortality in infected *Dif* ^−^ may partly be an artifact of this mutant’s low longevity. We found that the mean lifespan of *Dif*^−^ (51.17 days) was not significantly different from that of the background CNBW (46.13 days) (*t* = 1.45, *p* = 0.15); therefore, longevity should not have constrained our results. The disparity in the survival of their mutants indicates that the high susceptibility of flies lacking Psh is not necessarily linked to *Dif* for most *Metarhizium* infections despite their being in the same pathway.

**Figure 7.**
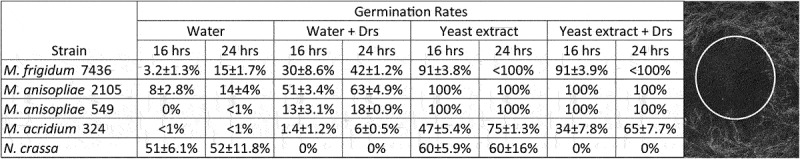
The effect of drosomycin on *Metarhizium.* (a) percent germination of spores of *Metarhizium* spp in water or yeast extract plus or minus drosomycin. (b) *N. crassa* on agar showing growth inhibition by 0.01 µg drosomycin applied to center of plate.

Persephone activates the späetzle-processing enzyme (SPE), which processes the extracellular Toll ligand späetzle (spz) [40] (Figure 5). We analyzed the role of these genes in resistance to Bb 80.2, and two representative pathogenic *Metarhizium*’s, *M. frigidum* (Mf7436) and *M. anisopliae* (Ma549). Flies lacking *Grass* (functions upstream of SPE), *SPE* or *spz* were significantly more susceptible to *Metarhizium* spp. and *B. bassiana* Bb 80.2 than their backgrounds. The survival time of *spz*^*rm7*^ mutants was reduced by 40% to 60% (depending on the fungal strain), similar to *psh^−^* flies. The antifungal peptide drosomycin is a product of *Dif* [40]. *Metarhizium* spores (Mf7436, Ma2105, Ma549 and Mac324) germinated at a higher frequency in aqueous solutions of drosomycin than in water alone (Figure 7), suggesting that *Metarhizium* spp. can use drosomycin as a nutrient source. Combining the antimicrobial peptides metchnikowin and cecropin with drosomycin had no additional impact on *Metarhizium* spores compared with drosomycin alone. The saprophytic non-pathogenic fungus *Neurospora crassa* used as a control was strongly inhibited by drosomycin (Figure 7).

Toll has also been reported to mediate the expression of Bomanins (Boms), a family of a dozen secreted peptides [41]. Boms are believed to be directly antifungal, even though synthetic Bom peptides lack *in vitro* antifungal activity [42]. Ten of the 12 *Bom* genes are tandemly arrayed in a cluster, and a mutant (Bom∆55C) lacking this cluster was almost as susceptible to *Metarhizium* spp. as *Psh ^−^* flies (Figure 6). Similar results have been reported for septic wounding with bacteria and a non-entomopathogenic filamentous fungus, suggesting that the loss of the 55C *Bom* cluster is highly detrimental to defense [41].

**Figure 8.**
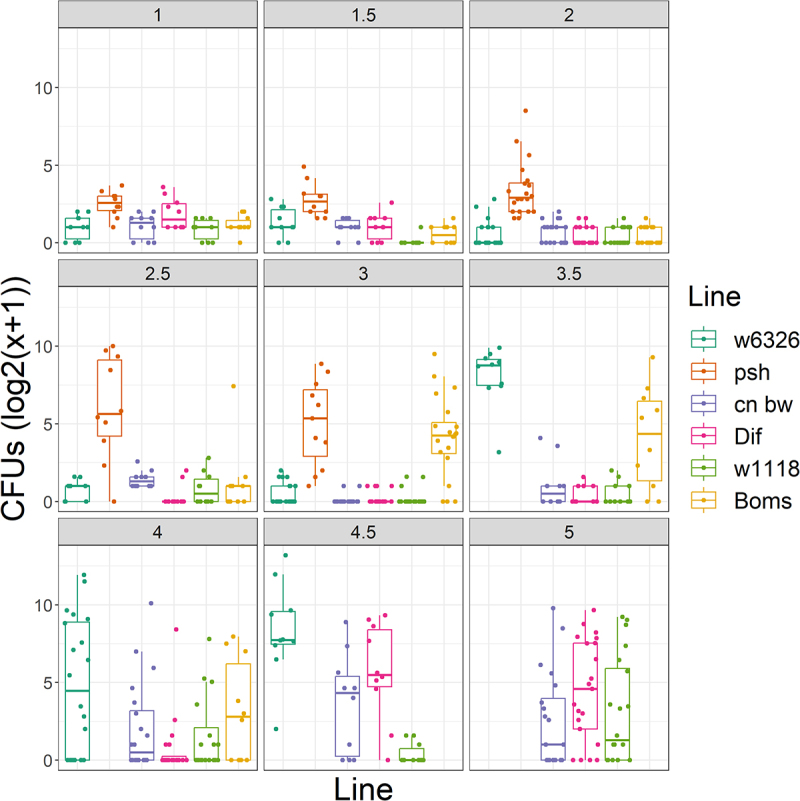
Time course of Ma549 fungal loads in the hemolymph of select immune mutants and their corresponding isogenic WT backgrounds. Results are presented as box plots.

To discriminate between disease (fungal load) and death, we assayed fungal load over the course of Ma549 infection, using wild-type, *Dif* ^−^, *Psh*^−^, and *Bom*^*Δ55C*^ flies in parallel (Figure 8). Because Ma549 infected *Bom*^*Δ55C*^ and *Psh*^−^ flies had a median survival of about 3.5 days, time points were taken at intervals of up to 4 days for these lines. Fungal loads in the three lines of WT flies only climbed rapidly when flies were close to death, indicative of resistance breaking down (Figure 8). In contrast, CFUs were elevated 48–72 h post-infection in *Bom*^*Δ55C*^ and *Psh*^−^ flies, so that at 72 h *Bom*^*Δ55C*^ flies averaged 75 CFUs compared to 0.25 in the wild-type. The fungal load of *Psh*^−^ flies was similarly elevated relative to that of wild-type flies. This early elevation in fungal load in *Psh*^−^ and *Bom*^*Δ55C*^ flies suggests that *Psh*^−^ signaling, in general, and Bom peptides specifically, contribute to resistance against Ma549. The appearance of CFUs 1.5 days post-infection in *Psh*^−^ flies precedes detectable Drosomycin-fluorescence in Drs-GFP flies by about half a day and perhaps represents a more accurate estimate of the speed of crossing of the integument.

Toll has been reported to be responsible for sexual dimorphism in longevity in flies infected with bacteria and *B. bassiana* [43,44]. We previously found that males in most DGRP lines are more resistant to Ma549 than their female counterparts [7], and this was true for four of the five isogenic background strains deployed in this study. Exceptionally, female w[1118]^DrosDel^ flies, the background of *SPE* ^*SK6*^, *Relish* ^*E20*^ and *spz*^*rm7*^, lived longer than males following infection with Mf7436 and *B. bassiana* (*t* > 3, *p* < 0.05). Female w[1118]^DrosDel^ flies infected with Ma549 also lived longer than males, but this was not statistically significant (*t* = 2.25, *p* = 0.07). Most of these mutations reverse the direction of dimorphism so that w[1118]^DrosDel^ becomes like most other fly lines with males more resistant than females. Flies lacking peptidoglycan recognition protein SA (PGRP-SA) implicated in the recognition of Gram-positive bacteria, did not affect the susceptibility of females to fungi, but male *PGRP-SA*^*seml*^ was significantly more resistant to Ma549 (*t* = 7.22, *p* = 0.0001) and *B. bassiana* (*t* = 8.94, *p* = 3.29E–5). *Psh^Δ^* flies infected with *M. frigidum* (*t* = 3.46, p*=*0.01) retained significant sexual dimorphism, unlike *psh^Δ^* (*t* = 1.58, *p* = 0.16) flies infected with Ma549.

The Imd pathway is not involved in the detection of a fungal infection, but downstream crosstalk with the Toll pathway has been suggested previously; in particular, the survival of Imd pathway mutants against *B. bassiana* is lower than that of the wild-type [33,45]. In our study, flies mutated in *Relish*, the terminal transcription factor in the Imd pathway, but not *Imd* itself, showed variably reduced survival, ranging from 15% (females infected with Ma549) to 37% (females infected with Mf7436). As reported by Shahrestani et al. [43], we also found that disrupting *Relish* eliminated sexual dimorphism in the survival of *B. bassiana* (*t* = 0.67, *p* = 0.52). The 37% reduction in female longevity compared to the background w[1118]^DrosDel^ was sufficient to reverse sexual dimorphism in response to *M. frigidum* infection so that males lived longer than females (Figure 6). These results suggest that the immune sexual dimorphism common in *Drosophila* lines is dependent on the specific interactions of each pathogen with immune pathways that contain some components (e.g., PGRP-SA) differentially affecting survival of males and other components (e.g., Relish) differentially affecting survival of females, at least in some lines and to some infections.

## Discussion

There could be a fitness advantage to higher virulence for an entomopathogen with an insect host that must be quickly disabled before it dies from other causes that will likely also kill the pathogen [10]. The observable variations in pathogenicity shown by *Metarhizium* species are, therefore, likely maintained by more complicated evolutionary processes. Classic evolution of virulence theory is based on trade-offs between pathogen growth, transmission, and host survival, which predicts that by extracting more resources from the host, the pathogen will grow faster and produce more infectious propagules [46]. However, experimental data for numerous pathogens show the opposite correlation, with slower growing pathogens being more virulent than faster growing ones [46]. Insect pathosystems involving fungi also differ from those commonly treated in traditional models in that sporulation of entomopathogens only occurs after host death, so that host death may increase pathogen fitness by allowing transmission. This is exemplified by a strain-like Mr2575, which kills the host with toxins and then colonizes post-mortem [15]. Despite this, our data showed that fast growth on PDA was associated with rapid kill (*r*≤-0.65, p ≤ 0.006) and sporulation (r = 0.49, p = 0.0531). Thus, although *Metarhizium* species possess a broad spectrum of mechanisms of pathogenesis (e.g. only some broad-host-range strains produce toxins that could require a high investment of resources), they do not swamp a simple non-host-specific relationship between the growth potential of mycelia and virulence. However, the strains that exhibited lower virulence and mycelial growth on PDA produced greater fungal loads in the hemolymph preceding or during the immobilization period and also induced late expression of drosomycin. In contrast to proliferation in the hemolymph, saprophytic growth on PDA was significantly correlated with rapid germination on fly wings (r = 0.67, p = 0.0046), suggesting that 1) radial growth on agar is a measure of potential virulence because it predicts rapid growth on host surfaces and 2) faster growth through the cuticle by virulent strains induces early expression of the Toll pathway. For non-toxin-producing strains (e.g. most specialists), variable numbers of fungal cells produced by different strains within the insect influence the rate of killing. A previous screen of mutant *Drosophila* lines found that greater growth of Ma549 (a generalist strain that produces few toxins) within the host was correlated with shorter life spans and earlier onset of sporulation (63.7% of the variation in life span was explained by variation in fungal load) [30].

For some bacteria that kill insects by bacteremia, LT_50_ values also directly correlate with *in vivo* growth rates [47]. Although such a quantitative relationship is not unexpected for an entomopathogen, a few studies on *B. bassiana* have not found correlations between virulence and *in vitro* growth rate [48,49]. Such associations appear to be limited to some plant pathogenic fungi [50], including *Alternaria brassicicola* where *in vitro* growth rate was weakly correlated with aggressiveness to its host [51]. These authors also found a trade-off between growth and spore production, such that faster growing isolates produced fewer spores, and postulated that such trade-offs might contribute to the maintenance of variation in pathogen populations. We did not detect similar trade-offs with fast-growing *Metarhizium* strains after host death and sporulation; instead, trade-offs seemed more likely to affect latent periods, which are longer for less virulent pathogens. Complicating interpretations of these data, the forces generating diversity within *Metarhizium* spp. will likely reflect adaptation to multiple environments, including plant roots, for at least some generalists such as Mr2575 [10]. It has been suggested that the ability to infect a phylogenetically broad range of hosts may have evolved to maximize protection to the plant (the permanent home of the fungus as distinct from the ephemeral insect) and provision of nutrients to the plant from cadavers via the fungus [10]. Thus, the virulence of plant-colonizing broad-host-range strains to insects is likely to evolve depending on any ways in which virulence affects changes in transmission to plants.

Specialization to particular hosts can be qualitative, characterized by the inability of a pathogenic isolate to infect many hosts, or quantitative, where the pathogens have lower performance [38]. The specialization of most *Metarhizium* strains appears to be quantitative, as they kill fruit flies, albeit slowly. Compared to most generalists, specialists also showed lower rates of *in vitro* mycelial growth and were less nutritionally flexible, that is, little growth on 0.01% YEM and glucose. This is consistent with more limited ecological associations with plants and as saprophytes and poor germination on cuticle. However, the hemipteran specialist *M. album* 1941 was not able to cause disease even in flies with impaired immunity. The other *Metarhizium* strains killed normal hosts, and, except for Mp443, this was amplified when the immune system was compromised.

Most *Metarhizium* species that readily kill multiple orders of insects also produce toxins, whereas the nontoxigenic *Metarhizium* spp. (e.g. *M. acridum, M. majus, M. album*) have narrow host ranges [15]. There are exceptions to this: the broad host range Ma549 does not produce much destruxins (Dtx) *in insecta* [24] [15]. The specialists had a notably long “immobilized time” compared to the generalists, which commenced with the appearance of numerous fungal propagules in the hemolymph. However, disrupting Dtx in Mr2575 did not significantly lengthen the immobilization time, or as previously reported longevity [39], suggesting that Dtx production by itself is not the time limiting factor for either pathogenic parameter.

Flies disrupted in *Psh* or *Spz* were similarly highly susceptible consistent with the so called danger arm of the Toll pathway being the principal one activated [28]. However, disrupting the downstream *Dif* resulted in only a small and statistically insignificant increase in susceptibility to Ma549 [39]. We found that the impact of *Dif* ^−^ on *B. bassiana* was significant but much less than that of disrupting *Psh*. *B. bassiana* kills more slowly than Ma549 and most other generalist *Metarhizium* strains, but it is unlikely that the apparent lack of impact of *Dif* ^−^ on Ma549 was due to the rapid lethality of *M. anisopliae*, as *Dif* ^−^ did not significantly increase the susceptibility to low-virulence *Metarhizium* isolates, unlike *Psh*^−^. An interesting exception was provided by the increased susceptibility of *Dif* ^−^ to *M. frigidum* 7437, which, like *B. bassiana* is a broad host range strain but kills comparatively slowly (Figure 6). A potential complicating factor is the possibility of partial redundancy between Dif and its paralog, the neighboring dorsal gene, but this is only reported to affect the induction of drosomycin at the larval stage [52].

*Beauveria* spp. evolved into insect pathogens independent of the *Metarhizium* lineage, and similar expansion of protease, chitinase families, etc., reflects the convergent evolution of an “entomopathogenicity toolkit” associated with functions necessary for insect pathogenesis [53]. As *Metarhizium* and *Beauveria* inevitably confront the insect immune system, they independently evolved a series of strategies to evade or overcome these immune responses. There are features unique to *Metarhizium* spp., which include blastospores producing a collagen coat (MCL1) to mask antigenic cell wall β-glucans from phagocytes [19], as well as destruxins [15,35,54]. Despite this, several generalist *Metarhizium* species evoke a rapid and robust innate immune response, showing that they do not escape recognition or block activation. A cost of opportunistically infecting multiple-host species may be that a generalist *Metarhizium* spp. cannot adapt to the immune system of each potential host. *Metarhizium* like *Beauveria* [55] has evolved resistance to the defensin-like peptide drosomycin, presumably under strong long-term selective pressure. Defensins are ubiquitous in arthropods, and *Metarhizium* can be engineered to express large quantities of scorpine (from the scorpion *Pandinus imperator*), a structurally similar but more potent antifungal/protozoan than drosomycin, with no harm to itself [56]. In contrast to defensins, Bom peptides are unique to *Drosophila* spp [41]. They may have evolved in response to the selective pressure exerted by entomopathogenic fungi after they became resistant to defensins. Similarly, Gottar *et al.*, (2006) [29] speculated that the *Psh*-dependent system evolved after the *GNBP*-3-based fungal cell-wall sensing system. *Metarhizium* may have evolved ways to escape ancient widely distributed defenses, including the effects of *Dif* activation and drosomycin, but not more recent insect innovations such as *Psh* activation and phylogenetically restricted Bomanins.

## Materials and methods

*Fungal strains. Beauveria bassiana* 80.2 (Bb80.2) was kindly donated by George Dimopoulos (Johns Hopkins Bloomberg School of Public Health). This *B. bassiana* strain has been used as a representative fungal pathogen in *Drosophila* studies [57]. We validated the species identification as *B. bassiana* by BLASTing the sequencing results of the *Tef-*1α region after PCR amplification using primers F:ATGGGTAAGGACGACAAGAC and R:GGAAGTACCAGTGATCATGTT. *M. robertsii* ARSEF 14447 was isolated from a female Spotted Wing *Drosophila* (*Drosophila suzukii*) collected by the first author, Dr Jonathan Wang, at a fruit farm in Keedysville, Maryland. Other fungal strains were obtained from the USDA Entomopathogenic Fungus Collection (Ithaca, N.Y., USA). The strains used were *M. anisopliae* (generalists 2105, 7427, and 549), *M. robertsii* (generalists 2575 and 1046), *M. brunneum* (generalist 346), *M. frigidum* (generalists 4124, 7436), *M. pingshaeaense* (generalists 538, 2162, gryllid specialist 443), *M. majus* (scarab specialists 297, 1946), *M. acridum* (acridid specialists 5736, 324), and *M. album* (hemipteran specialist 1941) (see S Table 1 for the origin of the strains). These fungal cultures were moved from −80°C stock tubes 10–14 days before each bioassay and grown on potato dextrose agar (PDA) at 27°C. Infection structures (appressoria) were induced by germinating conidia in yeast extract medium (YEM, 0.0125%) in polystyrene petri dishes, as described previously [58]. Alternatively, to test the isolates’ nutritional requirements for germination and differentiation, conidia were germinated in 0.1% YEM or in glucose medium (1% glucose, 0.1% NaNO_3_, 0.05% KH_2_PO_4_, 0.05% MgSO_4_) as previously described [23]. To facilitate studies of strain differences, we transformed several *Metarhizium* strains to express green fluorescent protein (GFP). Plasmid construction and transformation for GFP fluorescence were performed as described previously, and transformants were selected based on WT growth in culture and WT levels of virulence [59].

*Fly strains and infection protocols*. *Bom*^*Δ55C*^ was kindly donated by Steven Wasserman (University of California, San Diego, CA, USA). Drs-GFP, *Dif*^*1*^, *psh^Δ^* and their isogenic control lines were kindly donated by Dominique Ferrandon (University of Strasbourg) [60]. *PGRP-SA*^*seml*^, *SPE* ^*SK6*^, *Grass*^*Herrade*^, *Relish* ^*E20*^, and *spatzle*^*rm7*^ mutants and their isogenic control lines were kindly donated by Bruno Lemaitre (École Polytechnique Fédérale de Lausanne, Lausanne, France). Most of the mutants had w[1118] backgrounds, but we found that w[1118] from different sources differed slightly but consistently in susceptibility to *Metarhizium* spp. and *B. bassiana*, and are distinguished here as w[1118]^6326^, w[1118]^VDRC^ and w[1118]^DrosDel^ (S3 Table). The IMD (17474) mutant line was obtained from the Bloomington *Drosophila* Stock Center (flystocks.bio.indiana.edu/). We sequenced both *Dif*^*1*^ and its isogenic control (cn bw) to confirm that guanine 1104 (found in the cn bw) was point-mutated into adenine, resulting in a radical missense change from glycine to aspartic acid in *Dif*^*1*^.

For infection bioassays, *Metarhizium* strains were used, as described previously for Ma549 [7]. Fungal cultures were thawed 10–14 days before each bioassay from −80°C stock vials and grown on potato dextrose agar media plates at 27°C. Drs-GFP flies 2–4-days old were assayed at a final spore concentration of 1 × 10^4^ to 1 × 10^6^ (2.5 × 10^4^ for mutant studies) conidia/ml. Infected flies were maintained at 27°C and ~85% relative humidity on standard cornmeal molasses medium without tegosept or propionic acid.

To prepare the inoculum, conidia were suspended in sterile-distilled water, vortexed for 2 min, and filtered through Miracloth (22–25 µm) (Andwin Scientific) to remove mycelia. Spore concentrations were determined using a hemocytometer and were adjusted with water. Flies were vortexed with 20 mL spore suspensions for 30 s, collected by filtering the suspensions through Miracloth, and transferred to vials containing fresh food. Less than 10% of flies vortexed with water alone (mock-infected) or conidial suspensions died within 1 day, with no significant differences between lines, so flies succumbing within 1-day post-infection were deleted from the infection data. Following infection, flies were examined at six-hour intervals to determine the time to immobilization (flies not walking but still responding to touch) and time to death (completely moribund). Survival was monitored after topical inoculation in groups with at least three replicates (20–30 flies each) per sex per line. The number of dead flies was recorded twice per day for 10–14 days and the LT50 values (lethal time in days at which 50% of the flies died) were calculated using R.

Drosomycin reporter Drs-GFP flies were used to check for temporal differences in immune-response fluorescence. In a previous study, we reported that Dif^−^ mutant Drs-GFP flies showed greatly reduced expression of *Drs* GFP fluorescence in response to Ma549, which was confirmed by real time-PCR [30], suggesting that *Drs-*GFP provides a true readout of activation of the Toll pathway in response to *Metarhizium*. The fluorescence of 10 individual flies per time interval per infection with a 1 × 10^5^ spore suspension of each *Metarhizium* strain was quantified using a FilterMax F5 microplate reader. Data were collected for up to 10 days post-infection for the less virulent strains (Mp443, Ma324, *M. album* 1941).

We used previously described protocols for the bioassay of fungal growth in the hemolymph [30]. At each time point, 10 flies of each sex were individually homogenized with 45 μl of 0.1% Tween 80. The homogenate was spread onto Rose Bengal Agar plates supplemented with ox bile, oxytetracycline, streptomycin, penicillin, and chloramphenicol. Colony-forming units (CFUs) were counted after 7–10 days of incubation at 25°C.

To determine the effect of destruxin (Dtx) on the immobilization period of ~10 female Drs-GFP flies, they were infected with either Ma549, Mr2575, or Mr2575ΔDtx, collected without anesthesia, placed into food vials, and monitored at 3 h. intervals until death. Germination rates of cuticles and fungal growth in the hemolymph by GFP-tagged *Metarhizium* strains infecting w[1118]^DrosDel^ *Drosophila* were monitored as previously described [30]. For each fly, we evaluated conidia on the tergum and wings in four abdominal intersegmental regions and six dorsal and ventral areas on the abdominal segments.

We tested the effects of different (98% and 80%) relative humidity (RH) levels on infection parameters of a virulent *Metarhizium anisopliae* strain (Ma2105) isolated from *Hydrellia sp*. [Ephydridae; close relation to *Drosophila*], and an *M. pingshaense* strain (Mp443) with a strong host preference for crickets (Gryllidae) and low virulence in *Drosophila*. Female w[1118]^DrosDel^ flies were infected with GFP-fluorescent Mp443-GFP or Ma2105-GFP, and spore germination rates and hyphal lengths were monitored microscopically post-infection at different RH. RH was measured using a traceable Digital Humidity/Temperature Meter (Fisher Scientific™). Ninety-eight % and 80% RH were achieved using ddH_2_0 soaked paper towels and highly concentrated NaCl, respectively. These liquids were placed into a plastic Tupper ware box that contained a support for Petri dishes containing flies. Control or infected flies were briefly immobilized with CO_2_ and gently tapped into Petri dishes. They were localized to each dish with an autoclaved nylon mesh stretched over the dish and sealed with the edges of a lid, in which the face had been removed. Approximately 1 g of the cornmeal molasses diet was placed on the mesh, and flies were free to walk over the mesh to feed (Figure 2).

### Post-mortem analysis

The ability of the different fungal strains to colonize and exploit *Drosophila* cadavers was measured. For the emergent period, latent period, and sporulation capacity, 10 female flies harvested within 6 h of death were individually transferred into tubes containing a damp cotton ball. At 6-h intervals, we recorded the interval between death and emergent hyphae covering at least half of the fly cadaver (emergent period) and the appearance of spores (latent period). After 20 days, 500 μl of 0.1% Tween 80 was added to each tube, the tubes were vortexed (1 min), and spores per individual fly were counted using a hemocytometer (sporulation capacity). The results represent the average of 10 flies per fungal strain. Correlations between LT_50_ survival values, emergent period, sporulation capacity, and immobilization time were analyzed using GraphPad Prism 7 (GraphPad Software, Inc.) or R.

### Analysis of immune peptides

The expression and purification of drosomycin was performed as previously described [61]. The sequence encoding the mature drosomycin (Drs) was amplified via PCR from *D. melanogaster* genomic DNA and cloned into the NcoI and BamHI sites of a pET-32b expression vector derivative used for transformation of *Escherichia coli* strain Rosetta-gami (Novagen). Recombinant drosomycin, fused to a His_6_ tag, was purified on a HisTrap® affinity column (GE Healthcare) and the tag was cleaved with thrombin. Drosomycin was purified using a Resource® 3-ml reverse phase high-pressure liquid chromatography column. The molecular mass of recombinant drosomycin was confirmed by mass spectroscopy. Cecropin A was purchased from Sigma-Aldrich (St. Louis, MO, USA). Metchnikowin was synthesized as a service by Peptide 2.0, Inc. (Chantilly, VA). The effect of peptides on different fungi was determined by adding 50 µL of peptide (0.5 mg/ml) to 60 µL of water or 0.2% yeast extract containing *~*1 × 10^5^ fungal spores/mL, and calculating the percentage of germinated spores at 16 and 24 h.

## Supplementary Material

Supplemental Material

## Data Availability

The authors confirm that data supporting the findings of this study are available within the article and its supplementary materials.
